# Gene Therapy for Lipid Management: Dawn of a New Era

**DOI:** 10.7759/cureus.112496

**Published:** 2026-07-11

**Authors:** Prabhash C Manoria

**Affiliations:** 1 Cardiology, Manoria Heart and Critical Care Hospital, Bhopal, IND

**Keywords:** atherosclerotic cardiovascular disease, dyslipidemia, lipoprotein(a), low-density lipoprotein cholesterol, rna interference

## Abstract

Dyslipidemia is a major driver of atherosclerotic cardiovascular disease, with elevated low-density lipoprotein cholesterol (LDL-C) and triglycerides playing key roles in disease progression. Although conventional lipid-lowering therapies such as statins, ezetimibe, bempedoic acid, and proprotein convertase subtilisin/kexin type 9 inhibitors are effective, their impact is often limited by lifelong treatment requirements, suboptimal adherence, and inadequate achievement of lipid targets, particularly in patients with inherited disorders such as familial hypercholesterolemia. Recent advances in gene-based therapies have introduced a paradigm shift by targeting lipid metabolism at its genetic and molecular basis. Techniques including CRISPR-Cas9 gene editing, small interfering RNA, and antisense oligonucleotides enable precise modulation of key genes such as *PCSK9*, *ANGPTL3*, *APOC3*, and *LPA*, leading to sustained reductions in atherogenic lipids. These therapies act upstream at the level of gene expression or mRNA, offering improved specificity, longer duration of action, and the potential for infrequent or single-dose administration. Clinical and translational studies demonstrate significant lipid-lowering efficacy across multiple pathways, including LDL-C, triglycerides, and lipoprotein(a), addressing previously unmet therapeutic needs. Despite their promise, important challenges remain, including long-term safety concerns, off-target effects, cost, ethical considerations, and regulatory complexities. Emerging strategies such as combination gene targeting and personalized genomic therapy further expand therapeutic potential while supporting a transition toward preventive cardiology. Overall, gene-based therapies represent a transformative approach in lipid management, with the potential to overcome the limitations of conventional treatments and enable durable, precision-based cardiovascular risk reduction.

## Introduction and background

Dyslipidemia represents a key determinant of atherosclerotic cardiovascular disease (ASCVD), with increased levels of low-density lipoprotein cholesterol (LDL-C) and triglycerides serving as principal contributors to atherogenesis and subsequent cardiovascular (CV) events [1]. According to the Metabolic Non-communicable Disease Health Report of India, the prevalence of dyslipidemia was 81.2% [2]. Globally, approximately one in three adults has hypercholesterolemia, contributing substantially to coronary events and cerebrovascular accidents. The reported global prevalence of hypertriglyceridemia, hypercholesterolemia, low high-density lipoprotein cholesterol (HDL-C), and high LDL-C is 28.8%, 24.1%, 38.4%, and 18.93%, respectively [3].

Contemporary clinical management has predominantly emphasized lipid reduction through pharmacological therapies, including statins, ezetimibe, bempedoic acid, and proprotein convertase subtilisin/kexin type 9 (PCSK9) inhibitors, all of which have demonstrated substantial efficacy in lowering CV risk across diverse patient populations. Nevertheless, these approaches primarily target circulating lipid parameters and necessitate prolonged, often lifelong, administration to sustain their therapeutic effects [1,4].

Although established first-line treatments such as statins, along with newer agents such as PCSK9 monoclonal antibodies, have proven effective in attenuating the progression of coronary artery disease in a significant proportion of patients, a notable subset derives limited benefit. This reduced effectiveness may arise from multiple factors, including drug intolerance, concerns regarding adverse effects, genetic variability influencing drug responsiveness, and poor adherence associated with prior negative treatment experiences or reluctance to continue long-term pharmacotherapy [1]. These limitations are particularly evident in individuals with inherited lipid disorders, such as familial hypercholesterolemia (FH), in which genetic defects disrupt lipid homeostasis, resulting in persistently elevated LDL-C concentrations from an early age [5]. Consequently, there remains a substantial unmet need for novel therapeutic strategies capable of achieving more robust risk reduction while alleviating the challenges associated with sustained medication use. In this context, emerging technologies such as gene editing are beginning to offer promising alternatives, with the potential to deliver durable therapeutic effects through infrequent dosing, thereby improving patient outcomes and quality of life [1].

Gene therapy treats genetic disorders by introducing engineered genetic material into cells to restore or modify gene function. It primarily employs two approaches: gene addition, which introduces a functional copy of a defective or missing gene, and gene editing, which precisely modifies the genome to correct disease-causing mutations. Gene editing offers the potential for a durable, one-time treatment with long-lasting therapeutic benefits [6]. Advances in gene editing technologies and RNA-based therapeutics have introduced a paradigm-shifting approach by addressing lipid dysregulation at its underlying genetic basis. Techniques such as gene editing, RNA interference (RNAi), and antisense oligonucleotide (ASO) therapies are designed to alter or suppress the expression of critical genes involved in lipid metabolism, including *PCSK9*, angiopoietin-like protein 3 (*ANGPTL3*), apolipoprotein C3 (*APOC3*), and lipoprotein(a) (*LPA*). By targeting these molecular pathways, these interventions can produce sustained and potentially long-lasting reductions in atherogenic lipid levels [1,4]. The APOC3, a liver-derived glycoprotein, regulates triglyceride metabolism by increasing very-low-density lipoprotein production, inhibiting lipoprotein lipase activity, and reducing hepatic clearance of triglyceride-rich lipoproteins. Genetic studies have consistently shown that loss-of-function mutations in *APOC3* are associated with approximately 40% lower triglyceride levels and a similar reduction in coronary heart disease risk, highlighting ApoC-III as an important therapeutic target [5,7]. Similarly, the *PCSK9* is a key regulator of LDL-C metabolism. It promotes lysosomal degradation of LDL receptors (LDLR), reducing hepatic LDL uptake and increasing circulating LDL-C levels. [5]. Gene-silencing approaches targeting *ApoB-100* and *PCSK9*, including ASOs and RNAi, have demonstrated significant LDL-C lowering, although early ApoB-directed therapies were limited by adverse effects. Other emerging targets include *ANGPTL3* and *LPA* [8]. The ANGPTL3 inhibits lipoprotein and endothelial lipases, and its inhibition through ASOs, small interfering RNA (siRNA) therapies, or monoclonal antibodies has shown favorable reductions in triglycerides and LDL-C. The LPA, an LDL-like particle containing apoLPA, is an independent genetic risk factor for CV disease and aortic valve calcification. Novel gene-silencing therapies targeting apo(a) have produced substantial reductions in LPA levels, highlighting their potential for CV risk reduction [5]. By mimicking naturally occurring loss-of-function genetic variants associated with reduced CV risk, such approaches provide the opportunity to transition lipid management from continuous pharmacological suppression to enduring biological modification following a single or limited number of treatments [1]. This emerging therapeutic landscape signifies a fundamental transition from conventional chronic drug-based management toward durable modification of disease pathways at the genomic level. Such a shift carries profound implications for both the prevention and treatment of CV disease. Successful integration into routine clinical practice will depend on a thorough understanding of the mechanistic foundations, therapeutic advantages, potential limitations, and safety considerations associated with these novel interventions, as well as their alignment with existing treatment frameworks. A comprehensive evaluation of this evolving field necessitates careful consideration of the shortcomings of current therapies, the principles underlying gene-based approaches, and the expanding body of clinical evidence supporting their potential role in CV care.

## Review

Current lipid-lowering therapies

Current guidelines recommend plasma LDL-C as the primary target for reducing CV risk, with lower thresholds for higher-risk individuals and a stepwise approach to therapy escalation. Statins remain the first-line treatment for patients at sufficient risk of ASCVD [9]. They inhibit 3-hydroxy-3-methylglutaryl coenzyme A (HMG-CoA) reductase, reducing hepatic cholesterol synthesis and increasing LDLR expression, thereby enhancing LDL-C clearance. Ezetimibe is recommended as second-line therapy, either alone or with statins. It inhibits intestinal cholesterol absorption and reduces CV events in proportion to LDL-C reduction and treatment duration. Comparable outcomes are observed when equivalent LDL-C reductions are achieved with high-intensity statins or statin-ezetimibe combination therapy. PCSK9 inhibitors are advised for patients at very high risk or with FH. By preventing PCSK9-mediated LDLR degradation, these therapies enhance LDL-C clearance [4,9,10]. Despite clear recommendations, real-world implementation remains suboptimal. Statin monotherapy accounts for ~84% of lipid-lowering therapy use, yet only about 33% of patients achieve LDL-C targets [4,11]. In FH, target achievement is even lower, with only a small proportion reaching recommended levels [4,12]. Combination therapy is underutilized, used in only 10-21% of patients, contributing to poor goal attainment, especially in those with high baseline LDL-C [4,11,12]. Factors driving this gap include cost, limited access, safety concerns, poor treatment adherence, and gaps in patient awareness and perception of therapy benefits.

Disadvantages of traditional lipid control

Despite significant advances in lipid-lowering therapies, a substantial unmet need persists in the prevention and management of ASCVD, largely driven by challenges in treatment adherence and suboptimal attainment of lipid targets [13]. Maintaining long-term therapy for asymptomatic conditions such as elevated LDL-C remains difficult for both patients and healthcare providers, resulting in increased rates of hospitalization and adverse CV events.

Current guidelines recommend stringent LDL-C targets, particularly for high-risk populations. Patients at very high CV risk are advised to achieve LDL-C levels ≤1.4 mmol/L (55 mg/dL), whereas those at high risk should target ≤1.8 mmol/L (70 mg/dL) [13,14]. Although modelling suggests that up to 86% of patients could reach these goals with combination therapy including high-dose statins, ezetimibe, and PCSK9 inhibitors, real-world outcomes remain markedly inferior. Registry data indicate that only approximately 2% of patients with FH (heterozygous FH (HeFH)) achieve recommended LDL-C targets, and even among those receiving intensive triple therapy, only about 45% attain guideline-recommended levels [14-16]. Real-world evidence further highlights low utilization and persistence of lipid-lowering therapies. Around 50% of ASCVD patients are not on statin therapy, and only about 2% of eligible individuals are treated with PCSK9 inhibitors. Additionally, up to 50% of patients discontinue CV medications within 12 months of initiation. As a result, only about 3% of patients with HeFH achieve LDL-C goals, underscoring the gap between clinical guidelines and actual practice [13].

Non-adherence to therapy is multifactorial, with patient-related factors being most significant. These include younger age, low awareness of disease severity, concerns regarding adverse effects, and perceptions about medication safety. Treatment-related factors such as complex dosing schedules, polypharmacy, and adverse drug reactions also contribute to reduced adherence. Additionally, system-level challenges, including cost, limited access to advanced therapies, and variability in healthcare delivery, further compound this issue [13,17]. Behavioral factors play a critical role in treatment persistence. Patient preference is strongly influenced by regimen simplicity, dosing frequency, perceived effectiveness, side effect profile, and cost considerations [17]. Survey data indicate that while a majority of patients prefer conventional oral therapies, a significant proportion expresses interest in single-dose, long-acting interventions such as gene editing therapies, reflecting a desire for more convenient and durable treatment options [13,17].

Overall, the limitations of current lipid-lowering therapies are characterized by poor adherence, underutilization of combination regimens, high discontinuation rates, and inadequate achievement of LDL-C targets. These challenges highlight the need for innovative therapeutic strategies that can provide sustained lipid control with reduced dependence on continuous medication use.

Concept: treat the gene, not the biomarker

Gene-based therapies represent a fundamentally distinct strategy for lipid management by targeting regulatory processes at the level of gene expression rather than intervening on downstream protein activity. Conventional lipid-lowering interventions, including statins, primarily function through inhibition of enzymatic pathways and modulation of protein function; however, such approaches may lack specificity and can be associated with off-target effects owing to their broader modes of action [5,18]. In contrast, gene-based interventions are specifically designed to directly regulate the expression of genes that govern lipid metabolism, either through suppression or enhancement of gene activity with a high degree of precision. This targeted approach facilitates modulation of critical regulators such as PCSK9, ApoB, ANGPTL3, LPA, and ApoC-III, all of which play essential roles in determining plasma lipid concentrations and CV risk [5]. Collectively, these genes are involved in key biological processes, including hepatic lipoprotein synthesis, receptor-mediated clearance of LDL particles, and the metabolism of triglyceride-rich lipoproteins, thereby governing both lipid accumulation and clearance within the circulation. By influencing these upstream regulatory mechanisms, gene-based therapies address the underlying biological drivers of dyslipidemia rather than merely its downstream manifestations [5,19,20].

A defining feature of this therapeutic approach lies in its upstream mechanism of action. Instead of inhibiting protein activity after synthesis, gene-based therapies act at the level of messenger RNA (mRNA), thereby preventing protein production. RNA-targeted platforms, such as ASOs and siRNA, exert their effects by binding to specific mRNA sequences, which leads to degradation of the transcript through RNase H-mediated cleavage and subsequent suppression of translation. This enables highly selective and sustained inhibition of pathogenic proteins at their source [5,21]. An additional advantage of these therapies is their capacity to achieve elevated intracellular concentrations within hepatocytes, the primary site of lipid metabolism. This targeted delivery enhances therapeutic efficacy while minimizing systemic exposure and reducing the likelihood of off-target effects. Furthermore, many of these agents are engineered to preferentially accumulate within the liver, thereby improving tissue specificity and optimizing overall treatment efficiency [5,22]. Importantly, gene-based approaches also enable access to therapeutic targets that have traditionally been considered “undruggable” using conventional small-molecule agents or monoclonal antibodies, particularly proteins that lack enzymatic activity or are structurally challenging to inhibit. This significantly broadens the range of modifiable pathways in lipid disorders, including those involving LPA and ApoB [5,18].

Looking ahead, future developments may extend beyond gene silencing strategies to include gene replacement and gene correction techniques aimed at restoring normal gene function in individuals with inherited lipid disorders. These approaches are designed to directly address the underlying genetic defects rather than modulate downstream biological pathways. In addition, genome editing platforms such as CRISPR-Cas9 offer the potential for precise and potentially permanent modification of target genes, enabling either disruption or correction of pro-atherogenic pathways at the DNA level [23].

Taken together, these innovations signify a paradigm shift in lipid management, transitioning from repeated pharmacological modulation of circulating lipid biomarkers toward precise and potentially durable modification of the genetic determinants that regulate lipid metabolism.

Key technologies

Gene therapy for lipid management encompasses multiple complementary technologies that act at different stages of gene regulation. Key approaches include CRISPR-Cas9 for permanent gene editing, siRNA for silencing gene expression, and ASOs for inhibiting protein production, each offering distinct mechanisms to achieve sustained lipid lowering [5].

CRISPR-Cas9 is a gene-editing platform that enables precise and permanent modification of genes involved in lipid metabolism [24]. It uses a single guide RNA to direct the Cas9 enzyme to a specific DNA sequence, where it creates a double-strand break that is repaired by error-prone non-homologous end joining, resulting in gene disruption and long-term silencing [5,24]. This approach has been applied to key lipid-regulating genes such as *PCSK9* and *ANGPTL3*, where natural loss-of-function mutations are associated with reduced LDL-C, triglycerides, and CV risk [24,25]. Preclinical studies show that editing these genes improves lipid clearance pathways and leads to sustained lipid reduction [25]. Early clinical data further support this concept, with CRISPR-based *ANGPTL3* editing demonstrating ~50% reduction in LDL-C and ~55% reduction in triglycerides after a single dose [26]. This highlights the potential for a one-time, durable treatment option for dyslipidemia [24,26]. Key CRISPR-Cas9based therapeutic candidates, including Verve-101 (HEART-1) and Verve-102 (HEART-2), along with other targets under evaluation, are summarized in Table 1 [27-29].

**Table 1:** CRISPR-Cas9 gene editing. HeFH = heterozygous familial hypercholesterolemia; HoFH = homozygous familial hypercholesterolemia

Target gene	Drug	Trial	Patient subset
*PCSK9* [27,28]	Verve-101 [27]	HEART-1	HeFH
	Verve-102 [28]	HEART-2	HeFH
*ANGPTL3* [29]	Under evaluation		HoFH
	Under evaluation		Mixed dyslipidemia

However, concerns remain regarding off-target effects and genomic instability due to double-strand DNA breaks, and ongoing advancements such as high-fidelity Cas9 aim to improve precision and safety [24].

The RNAi using siRNA is an emerging therapeutic approach that enables sequence-specific silencing of target genes at the mRNA level, thereby reducing protein production [5,30]. Mechanistically, siRNA consists of double-stranded RNA molecules that guide the RNA-induced silencing complex (RISC) to degrade complementary mRNA, resulting in targeted suppression of gene expression [30]. This strategy is particularly well-suited for lipid disorders, as it can selectively inhibit hepatic genes such as *PCSK9*, *ANGPTL3*, and *APOC3*, which play central roles in lipid metabolism. Clinical and meta-analytic evidence demonstrates that siRNA therapies produce dose-dependent lipid reductions that vary by target gene, with *PCSK9*-targeting agents such as inclisiran showing substantial LDL-C lowering, while *APOC3*- and *ANGPTL3*-targeting therapies show stronger triglyceride reduction [30,31]. Preclinical and translational studies further support durable lipid lowering, with *ANGPTL3*-targeted siRNA showing prolonged reductions in triglycerides and non-HDL-C through sustained mRNA suppression [32]. A major advantage of siRNA therapies is their prolonged duration of action, as demonstrated by *ANGPTL3*-targeting siRNA (ANGsiR10), which sustained triglyceride reduction for up to 14 weeks after a single dose, allowing extended dosing intervals; similarly, clinically available agents such as inclisiran can be administered every six months, and other siRNA therapies have reduced dosing frequency from weeks to months, thereby improving patient adherence compared with conventional therapies [30,32]. Additionally, advances such as GalNAc conjugation enhance liver-specific delivery and reduce systemic toxicity, improving safety and efficacy [31,32]. However, challenges remain, including potential off-target effects, immune activation, and delivery-related limitations that require further optimization [30].

ASOs are single-stranded, synthetic nucleic acid molecules (15-30 nucleotides) that bind to complementary sequences on target mRNA and inhibit protein production [33]. They function through two principal mechanisms: steric inhibition of translation and RNase H1-mediated degradation of target mRNA, resulting in reduced gene expression. Chemical modifications such as phosphorothioate backbones and GalNAc conjugation improve stability, plasma half-life, and targeted delivery to hepatocytes. ASOs target key regulators of lipid metabolism, including ApoB, APOC3, LPA, and ANGPTL3, enabling selective modulation of LDL and triglyceride pathways [33,34]. Clinical evidence demonstrates target-specific lipid effects. For example, mipomersen (ApoB-targeting ASO) significantly reduces LDL-C, with approximately 24% reduction at 26 weeks in the pediatric population in clinical trials [34,35]. In contrast, volanesorsen (APOC3-targeting ASO) produces marked triglyceride lowering, achieving 77% reduction in the APPROACH trial and up to 86% reduction in familial chylomicronemia syndrome patients, while IONIS‑APOCIII‑LRx shows dose-dependent triglyceride reductions of 23-60% [33]. In contrast, pelacarsen (Apo(a)-targeting ASO) primarily reduces LPA, with reductions up to 92% and minimal effect on LDL or triglycerides [33,34]. Key ASO therapies evaluated in clinical studies, including olezarsen (BALANCE, CORE-TIMI 72A/72B), volanesorsen (APPROACH, COMPASS), and pelacarsen (HORIZON), are summarized in Table 2 [33,36-38].

**Table 2:** ASO-based gene therapy evaluated for lipid management. ASO = antisense oligonucleotide; FCS = familial chylomicronemia syndrome; SHTG = severe hypertriglyceridemia

Drug	Trial	Subset
Volanesorsen [33]	APPROACH	FCS and SHTG
	COMPASS	FCS and SHTG
Olezarsen [36]	BALANCE	FCS and SHTG
Olezarsen [37]	CORE-TIMI 72A	SHTG
	CORE-TIMI 72B	SHTG
Pelacarsen [38]	HORIZON	Increased LP(a)

ASOs offer high target specificity with dosing from weekly to monthly intervals, improving flexibility. However, injection-site reactions, thrombocytopenia, and liver-related adverse effects remain key limitations [33,34].

Gene therapy drugs in dyslipidemia management

Pelacarsen (Apo(a)-Targeting ASO)

Pelacarsen is a GalNAc-conjugated ASO that targets LPA mRNA in hepatocytes, leading to reduced Apo(a) synthesis [38,39]. Clinical studies show dose-dependent LPA reduction of ~35-80% [39], with higher reductions (~59-82% or ≥80% with repeated dosing) [38]. In the LPA FRONTIERS APHERESIS trial, pelacarsen achieved ~72% reduction in LPA and reduced the need for lipoprotein apheresis by ~99% [40]. Additionally, pelacarsen reduced oxidized phospholipids by 30-90% and LDL-C by ~20% in patients on background therapy [39]. It was well tolerated with no excess nephrotoxicity, though urinary tract infections were reported more frequently compared to placebo, and injection-site reactions remain the most common adverse event [39,40]. A large Phase 3 CV outcomes trial (lipoprotein(a) HORIZON) is ongoing to evaluate its impact on clinical outcomes [38,39].

Inclisiran (siRNA Therapy)

Inclisiran is a novel siRNA therapy that inhibits hepatic production of PCSK9, resulting in increased LDLR recycling and reduced LDL‑C levels. It is administered by subcutaneous injection on day 1, day 90, and every 6 months thereafter, enabling sustained lipid control. The twice‑yearly treatment frequency represents a major change from daily statin therapy or monthly PCSK9 monoclonal antibody injections, offering a simplified dosing strategy that improves long‑term management [41,42]. Inclisiran can reduce LDL‑C by ~50% and is used as an adjunct to maximally tolerated statin therapy in patients with ASCVD or HeFH, serving as an alternative to PCSK9 monoclonal antibodies [41]. The trials included patients with statin intolerance and homozygous familial hypercholesterolemia in ORION‑1 and ORION‑5, respectively, and demonstrated LDL‑C reductions of 40-52% compared to placebo over 540 days. Inclisiran also reduces PCSK9 (up to 80%), apoB (~36-48%), and LPA (~20%) [42]. It is well tolerated, with injection-site reactions as the most common adverse event, while overall adverse event rates are comparable to placebo [41-43]. The twice‑yearly dosing improves adherence and long-term lipid control, though CV outcomes trials (e.g., ORION‑4) are ongoing [42]. Inclisiran demonstrated a favorable safety profile, with no significant increase in all-cause mortality, major adverse cardiovascular events, diabetes risk, or respiratory adverse events. Injection-site reactions were the most common adverse effect but were generally mild, transient, and rarely led to treatment discontinuation, which suggests Inclisiran as a safe and well-tolerated long-term lipid-lowering therapy [44].

Other siRNA Therapies Targeting the LPA Gene

siRNA therapies targeting *LPA* represent a major advance in managing genetically driven CV risk. Key agents include olpasiran, lepodisiran, zerlasiran, and SLN360, which selectively silence hepatic *LPA* gene expression, thereby reducing Apo(a) production [30,31]. Clinical studies in hyperlipoproteinemia(a) show a mean reduction in apolipoprotein levels of 75.69%, with individual agents demonstrating dose‑dependent reductions. For instance, lepodisiran reduced apolipoprotein levels by 41% to 97% depending on dose, while zerlasiran achieved reductions of 30% to 90%. Similarly, SLN360 demonstrated reductions ranging from 46% to 98%, and olpasiran reduced apolipoprotein(a) by 66.9% to 97.5% across different dosing regimens. These effects are clinically significant given the lack of effective traditional therapies for LPA [30]. These agents are typically administered subcutaneously with infrequent dosing intervals, contributing to sustained lipid lowering. They are generally well tolerated, with common adverse events including injection-site reactions, nasopharyngitis, and mild systemic symptoms [30,31]. Key siRNA-based therapies evaluated in clinical studies, including plozasiran (PALISADE), olpasiran (OCEAN), lepodisiran (ACCLAIM), zerlasiran (ALPACAR), and inclisiran (ORION-9, 10, 11), are summarized in Table 3 [42,44-49].

**Table 3:** siRNA-based gene therapy evaluated for lipid management. *: Not an siRNA therapy but targets LPA. SHTG = severe hypertriglyceridemia; siRNA = small interfering RNA

Drug	Trial	Subset
Inclisiran [42]	ORION-9, 10, 11	PCSK9
Plozarsiran [45]	PALISADE	SHTG
Olpasiran [46]	OCEAN	LPA
Lepodisiran [47]	ACCLAIM	LPA
Zerlasiran [48]	ALPACAR	LPA
Muvalaplin (oral)* [49]	KRAKEN	LPA

Muvalaplin

Muvalaplin is an oral small‑molecule inhibitor of LPA formation that acts by disrupting the initial non-covalent interaction between Apo(a) and ApoB, thereby preventing assembly of the LPA particle [49,50]. Unlike RNA‑based therapies, it targets LPA assembly rather than Apo(a) production or *LPA* gene expression [49]. In clinical studies, muvalaplin has demonstrated dose‑dependent reductions in LPA, with a phase 1 trial showing maximum reductions of 63-65% after 14 days of dosing [50], and a phase 2 trial reporting placebo‑adjusted reductions of 47.6%, 81.7%, and 85.8% at doses of 10 mg, 60 mg, and 240 mg, respectively, using an intact LPA assay, with corresponding reductions of 40.4%, 70.0%, and 68.9% using an Apo(a)-based assay. At higher doses, a high proportion of patients achieved LPA levels below 125 nmol/L, demonstrating substantial lipid-lowering efficacy in high-risk populations [49].

Drugs Targeting the ANGPTL3/APOC3 Gene

*ANGPTL3* is a key regulator of lipid metabolism that acts by inhibiting lipoprotein lipase (LPL) and endothelial lipase, thereby increasing circulating triglycerides and atherogenic lipoproteins [51]. Inhibition of *ANGPTL3* enhances lipolysis and promotes clearance of triglyceride‑rich lipoproteins and LDL particles through LDLR-independent mechanisms, making it particularly useful in conditions such as mixed dyslipidemia, severe hypertriglyceridemia, and homozygous familial hypercholesterolemia [30,51]. RNA‑targeted therapies against *ANGPTL3* include siRNAs such as solbinsiran and zodasiran, which act by silencing hepatic *ANGPTL3* mRNA via the RISC, thereby preventing protein synthesis. Among RNA‑based therapies, solbinsiran (*ANGPTL3*‑targeting siRNA) suppresses hepatic *ANGPTL3* mRNA translation and has demonstrated dose‑dependent reductions in triglycerides up to ~73%, LDL-C up to ~30-42%, non‑HDL-C up to ~41-46%, and ApoB up to ~30-36% in clinical studies [51]. Similarly, zodasiran (ARO‑ANG3) has shown reductions in triglycerides of ~34-63%, ANGPTL3 levels up to ~70-74%, LDL-C up to ~20%, and non‑HDL-C up to ~29%, confirming robust lipid lowering in patients with mixed hyperlipidemia [30,51]. Key ASO and siRNA-based therapies targeting *ANGPTL3* and lipid pathways are summarized in Table 4 [51-54].

**Table 4:** Gene therapies targeting the ANGPTL3 gene. ASO = antisense oligonucleotide; HoFH = homozygous familial hypercholesterolemia; RNAi = RNA interference; siRNA = small interfering RNA

Drug	Trial	Subset
ASO		
Vupanorsen [52]	TRANSLATE-TIMI 70	Mixed dyslipidemia
RNAi (SiRNA)		
Solbinsiran [53]	PROLONG-ANG3 Phase-2	Mixed dyslipidemia
Zodasiran [51,54]	ARCHES-2b [51]	Mixed hyperlipidemia
	GATEWAY Phase-2 [51]	HoFH
	YOSEMITE Phase-3 ongoing [54]	HoFH

Targeting *APOC3* provides a complementary approach for hypertriglyceridemia. *APOC3* inhibits LPL activity and delays clearance of triglyceride‑rich lipoproteins. Its suppression enhances triglyceride metabolism [30]. The RNAi therapies, such as plozasiran (*APOC3*‑targeting siRNA) and ARO‑APOC3, have demonstrated marked reductions in triglycerides (~50-80%) and apolipoprotein levels (~23-88%), with consistent efficacy in hypertriglyceridemia and chylomicronemia syndromes. ASOs targeting *APOC3*, including volanesorsen and olezarsen, similarly reduce *APOC3* expression and triglyceride levels, further supporting this pathway as a key therapeutic target [30]. Similarly, *ANGPTL3*-targeting ASOs such as vupanorsen demonstrate a combined lipid-lowering profile, with triglyceride reductions of 33-63% and LDL-C reductions up to 33% depending on dose and regimen [33].

Challenges and concerns

Gene‑based therapies for lipid disorders face several important challenges and concerns that limit their clinical translation. Long‑term safety remains a major issue, particularly for genome editing approaches such as CRISPR‑Cas9, as these technologies introduce permanent DNA changes that cannot be reversed, necessitating careful and long‑term safety assessment and monitoring [55]. Off‑target gene effects represent another critical limitation, as CRISPR‑Cas9 and related platforms may interact with unintended genomic sites, leading to insertions, deletions, chromosomal rearrangements, or mutations in oncogenes or tumor suppressor genes [24,55]. In addition, extended expression of gene editing components can further increase off‑target activity, compounding safety risks [55]. Cost and accessibility also remain significant barriers, as gene therapies involve complex delivery systems, advanced manufacturing processes, and require specialized infrastructure, limiting widespread patient access [6,24]. Ethical concerns are particularly important due to the permanent and potentially heritable nature of genome modifications, raising questions about unintended long‑term consequences, germline transmission, and appropriate clinical boundaries for use in humans [24,55]. Finally, regulatory hurdles remain substantial, as these therapies require rigorous evaluation of safety, efficacy, delivery mechanisms, and long‑term outcomes, with evolving regulatory frameworks needed to address the complexity and novelty of gene editing technologies [24].

Future directions

Future directions in lipid gene therapy are rapidly evolving, with several promising developments highlighted across the literature. One major advance is the emergence of one‑time CRISPR‑based therapies targeting *PCSK9*, where early human trials and preclinical studies demonstrate that a single administration can achieve durable suppression of *PCSK9* and sustained reductions in LDL‑C, potentially eliminating the need for repeated dosing [24,56,57]. Additionally, there is growing interest in combination gene targeting strategies, where multiple lipid‑related genes such as *PCSK9*, *ANGPTL3*, *APOC3*, and *ApoB* are edited simultaneously or sequentially to address LDL‑C, triglycerides, and related pathways, with preclinical studies already demonstrating the feasibility of targeting multiple genes involved in dyslipidemia [56]. Meganucleases are sequence-specific deoxyribonucleases that recognize long DNA targets (14-40 bp) for gene editing. Although i-CreI-derived megacurases from *Chlamydomonas reinhardtii* are more challenging to engineer than CRISPR-Cas9, their high specificity and small size (~300 amino acids) make them promising gene-editing tools [58]. Another key direction is the move toward personalized genomic lipid therapy, as gene therapy approaches increasingly rely on identifying patient‑specific genetic variants and tailoring interventions to correct or modulate individual disease‑causing mutations, marking a shift toward precision medicine [24,57]. Although gene therapy represents a transformative approach to lipid management, its clinical impact will depend not only on efficacy and safety but also on equitable access. Socioeconomic factors such as income, education, insurance coverage, healthcare infrastructure, and geographic location substantially influence CV outcomes and access to advanced therapies. Individuals from lower socioeconomic backgrounds and underserved populations often experience a higher burden of dyslipidemia and ASCVD, yet may face barriers to receiving novel treatments due to cost, limited specialist availability, and inadequate healthcare coverage. Furthermore, disparities in health literacy and healthcare utilization may affect patient awareness, acceptance, and adherence to emerging genetic therapies. As gene-editing and RNA-based lipid-lowering interventions move toward clinical practice, strategies aimed at improving affordability, accessibility, and equitable distribution will be essential to ensure that the benefits of these innovations are realized across diverse populations rather than exacerbating existing CV health disparities [59]. Finally, these advances collectively support a broader shift toward preventive cardiology at the genetic level, where early genetic intervention, potentially even before disease onset, could modify lifelong CV risk by targeting the underlying molecular drivers, thereby transforming treatment from chronic management to long‑term prevention [56,57].

## Conclusions

Gene-based therapies represent a transformative shift in lipid management by targeting the genetic basis of dyslipidemia rather than simply lowering circulating lipid levels. The present study provides a comprehensive and translational perspective by integrating genetic insight, molecular mechanisms, and emerging technologies such as CRISPR-Cas9, siRNA, and ASO to achieve substantial and sustained reductions in LDL-C, triglycerides, and LPA, with the potential for infrequent or even one-time administration, addressing key limitations of traditional therapies such as poor adherence and incomplete target achievement. This review focused on the connection between naturally occurring genetic variants and the development of next-generation therapies targeting central regulators of lipid metabolism, including *PCSK9*, *ANGPTL3*, *APOC3*, and *LPA*. These therapies offer a more precise and comprehensive approach to lipid regulation while expanding treatment options for genetically driven conditions such as FH. Furthermore, the integration of these approaches into clinical care signals a broader transition toward personalized and preventive cardiology, where early intervention at the genetic level may alter lifelong CV risk. Nevertheless, despite their transformative potential, significant considerations remain regarding long-term safety, off-target effects, cost, accessibility, and regulatory complexity, underscoring the need for continued research and careful clinical integration as this field advances.
